# Evaluation of a Simple Clinical Language Paradigm With Respect to Sensory Independency, Functional Asymmetry, and Effective Connectivity

**DOI:** 10.3389/fnbeh.2022.806520

**Published:** 2022-03-03

**Authors:** Erik Rødland, Kathrine Midgaard Melleby, Karsten Specht

**Affiliations:** ^1^Department of Biological and Medical Psychology, University of Bergen, Bergen, Norway; ^2^Division of Psychiatry, Department of Child and Adolescent, Haukeland University Hospital, Bergen, Norway; ^3^Adult Habilitation Section, Telemark Hospital Skien, Skien, Norway; ^4^Mohn Medical Imaging and Visualization Centre, Haukeland University Hospital, Bergen, Norway; ^5^Department of Education, UiT The Arctic University of Norway, Tromsø, Norway

**Keywords:** functional magnetic resonance imaging, fMRI, language network, asymmetry, speech perception, clinical fMRI, reliability, dynamic causal modeling

## Abstract

The present study replicates a known visual language paradigm, and extends it to a paradigm that is independent from the sensory modality of the stimuli and, hence, could be administered either visually or aurally, such that both patients with limited sight or hearing could be examined. The stimuli were simple sentences, but required the subject not only to understand the content of the sentence but also to formulate a response that had a semantic relation to the content of the presented sentence. Thereby, this paradigm does not only test perception of the stimuli, but also to some extend sentence and semantic processing, and covert speech production within one task. When the sensory base-line condition was subtracted, both the auditory and visual version of the paradigm demonstrated a broadly overlapping and asymmetric network, comprising distinct areas of the left posterior temporal lobe, left inferior frontal areas, left precentral gyrus, and supplementary motor area. The consistency of activations and their asymmetry was evaluated with a conjunction analysis, probability maps, and intraclass correlation coefficients (ICC). This underlying network was further analyzed with dynamic causal modeling (DCM) to explore whether not only the same brain areas were involved, but also the network structure and information flow were the same between the sensory modalities. In conclusion, the paradigm reliably activated the most central parts of the speech and language network with a great consistency across subjects, and independently of whether the stimuli were administered aurally or visually. However, there was individual variability in the degree of functional asymmetry between the two sensory conditions.

## Introduction

### Presurgical Mapping of Language

Presurgical mapping of areas responsible for the production and comprehension of language is essential to minimize the risk of postoperative deficits in patients who receive surgery in the language dominant hemisphere. Over many decades, the presurgical mapping of language has been done using the Wada test (Benbadis et al., 1998; Wada and Rasmussen, 2007; Niskanen et al., 2012), but today the Wada test has mostly been replaced by more non-invasive methods, particularly neuroimaging. These methods offer much higher spatial resolutions than the near dichotomous descriptions resulting from the Wada test, and allow clinicians and researchers to shed light not only on which hemisphere is speech dominant, but also on the localization of specific speech and language functions and the connectivity between different neuroanatomical areas. Among the available non-invasive alternatives, functional magnetic resonance imaging (fMRI) is most dominantly used in those clinical examinations (Deppe et al., 2000; Jansen et al., 2006; Binder, 2011; Silva et al., 2018). However, it has also been reported that clinical fMRI might be better in identifying motor areas than language areas (Orringer et al., 2012).

Today, there is a plethora of different fMRI paradigms varying in design, method and efficacy described in the literature (Fernández et al., 2003; Benke et al., 2006; Tie et al., 2014; Urbach et al., 2015; Benjamin et al., 2017), including resting-state fMRI (Tie et al., 2014; Sair et al., 2016). Some of the more commonly used paradigms in presurgical language mapping involve simple word generation and reading tasks. These result in robust activation but do not fully cover the complex multimodal nature of language processing. For this reason, comparative studies recommend using several paradigms with different tasks and both visual and auditory stimuli to ensure robust activation and asymmetry measures of all the relevant areas (Engström et al., 2004; Niskanen et al., 2012). There is, however, no common consensus on the optimal combination of tasks for clinical presurgical mapping of language and hemispheric dominance. Using several paradigms also prolong the scanning procedure, which has its own list of drawbacks; ranging from the unpleasantness and impracticality of laying still inside a noisy MR scanner for long periods of time, reduced reliability due to varying degree of attention, to the inevitable higher economic cost connected to longer scan times. More effective language paradigms, which can create robust activations of relevant areas involved in both production and comprehension of language, and which are independent of whether visual or auditory stimuli have been used, is therefore to be desired. As the need of fMRI in clinical practice continues to expand (Specht, 2020), there is also a growing need for both aurally and visually mediated paradigms to accommodate for impairments of sight and hearing in the clinical population.

### The Neuroanatomy of Language

In order to develop a paradigm that activates the same speech and language areas with the same degree of hemispheric asymmetry, independent whether stimuli are presented visually or aurally, one has to operationalize current speech models into a multimodal paradigm. The classical Wernicke–Lichtheim model of speech processing, envisaged a simple network with a strong leftward asymmetry comprised of Broca’s area, responsible for the production of language; Wernicke’s area, responsible for language comprehension; and a third anatomically less specified area for the processing of concepts, as well as the connections between them (Shaw, 1874). While this early model is still taught, it is also well established that the Wernicke–Lichtheim model of speech processing is too simplistic and far from effective in describing the complexity of speech and their anatomical localization (Hickok and Poeppel, 2007; Specht, 2013, 2014). The traditional emphasis on the areas described by Paul Broca and Carl Wernicke has in later years given way to a more complex understanding of the language system. Current models recognize that both production and comprehension of language involve a widely distributed cortical network, and assume a more hierarchical structure with dynamic and context-dependent interactions between the different regions (Price, 2010, 2012).

One of the most influential models on the functional anatomy of language is the dual-stream-model (DSM), which postulates a ventral stream that is involved in speech comprehension and a dorsal stream that is involved in speech production. The ventral stream, which is partly bilaterally organized, provides important functions for basic speech perception, such as phonetic decoding, phonological and sub-lexical processing, and higher-order speech comprehension, like lexical, combinatorial and semantic processing, and appears to be organized along a gradient from posterior to anterior (Ueno et al., 2011; Specht, 2013). The dorsal stream, which is assumed to left lateralized, translates acoustic speech signals into articulatory representations and supports sensorimotor integration (Hickok and Poeppel, 2004, 2007; Poeppel and Hickok, 2004; McGettigan and Scott, 2012; Poeppel et al., 2012; Specht, 2014). There is also thought to exist a mostly left-hemispheric conceptual network, which is assumed to be widely distributed throughout the cortex. The two streams are considered to be hierarchically organized such that input to each processing step depends on the computational output of the previous step (Poeppel and Hickok, 2004; Hickok and Poeppel, 2007; Specht, 2013, 2014).

Anatomically, the early stages of speech processing are thought to occur bilaterally in the auditory regions of the dorsal superior temporal gyrus and superior temporal sulcus (Hickok and Poeppel, 2007; Specht, 2013, 2014). The two streams then emerge from the middle and posterior superior temporal sulcus, with the ventral stream spreading across structures in the superior and middle portions of the temporal lobe, and the dorsal stream comprising the premotor areas and the articulatory network, such as Broca’s area and the anterior insular (aIns) cortex (Specht, 2013). The ventral stream appears to be bilaterally organized, particularly at the phoneme-level of speech recognition, before forming a lateralization gradient from the posterior superior temporal lobe toward the anterior temporal lobe as the processing complexity increases. The dorsal stream does, however, appear to have a stronger left-sided lateralization (Hickok and Poeppel, 2000, 2004, 2007; Scott, 2000; McGettigan and Scott, 2012; Specht, 2013, 2014).

The dual stream model does not make any predictions when it comes to reading. On the other hand, it is known that, after the initial processing in the primary visual cortex, written letters and words are decoded by an area at the inferior border of the left occipital and left temporal lobe. This area is called the “visual word form area” and has strong connections to the left angular gyrus and into the speech and language network of the left temporal lobe (McCandliss et al., 2003; Dehaene and Cohen, 2011).

There are some further brain areas, which are not explicitly covered by the model but which nevertheless are repeatedly detected in neuroimaging studies (Price, 2012; Specht, 2014). Among the areas frequently identified in the studies covered by Price (2012) are the left angular gyrus and the left supramarginal gyrus. Put very simple and in the context of speech and language processes, these areas are typically associated with the semantic network, which is a wide-spread network and comprises besides these parts of the inferior parietal lobe, also areas of the temporal and frontal cortex, mostly of the left hemisphere (Binder et al., 2009; Fedorenko et al., 2011; Price, 2012; Graessner et al., 2021), and is broadly independent of the stimulus modality (Deniz et al., 2019). Similarly, the processing of sentences and syntax are assumed to take part in a network including the left posterior temporal lobe, the temporal pole and inferior frontal gyrus (IFG), and their degree of involvement in the processing might depend on syntactic complexity or predictability (Price, 2012; Matchin et al., 2017; Zaccarella and Friederici, 2017). Besides, outside of the classical language areas, also subcortical areas such as the basal ganglia, aIns, and cerebellum are repeatedly reported in neuroimaging studies (Price, 2012; Specht, 2014).

### Aim

This study aimed to develop further a paradigm for clinical use that reliably activates the language network in terms of brain activations, brain asymmetry, and effective connectivity, independent of whether the stimuli were delivered visually or aurally. The resulting auditory paradigm was based on a pre-existing visual paradigm that had already shown good potential in clinical practice (Berntsen et al., 2006). This paradigm presented the participants with tasks based on the popular television show “Jeopardy!”. In such a task, participants are presented with an answer and are required to respond by generating a corresponding question. Therefore, this task requires several processing steps that are distributed over different areas of the ventral and dorsal stream. First, the content of the sentence has to be semantically decoded. Second, an appropriate target word has to be retrieved from the lexicon. Third, a corresponding answer has to be formulated as a grammatically correct question. Fourth, the answer has to be articulated. This requires both semantic and lexical processing that are functions of the ventral stream, as well as the production of a corresponding sentence as a response that is a function of the dorsal stream. Moreover, these processes are assumed to be broadly independent of the sensory modality of the original stimuli to be processed.

We hypothesized that, irrespective of the sensory modality, this task activates large parts of the left hemispheric speech and language network, comprising the ventral stream for processing the stimuli and identifying the semantic content, and the dorsal stream for generating the appropriate response. More specifically, we expected activations in the superior temporal gyrus and sulcus, inferior parietal lobe, temporal pole, inferior frontal gyrus/frontal operculum, and parts of the articulatory motor network, including subcortical structures. Further, it was expected that the individual asymmetry of the activations is independent of the stimulus modality. Finally, it was predicted that the measures of effective connectivity are comparable between the two sensory stimulations, indicating that they both activate not only the same brain areas but the same network configuration.

To test that the described processes are independent of the sensory modality of the original stimulus that had to be processed, an auditory and a visual version of the jeopardy paradigm was created. Accordingly, it was hypothesized that both versions would activate the dorsal and ventral stream of the speech and language network to the same extent, and that the effective connectivity within the speech and language network will be unchanged.

## Materials and Methods

### Participants

Twenty-one healthy Norwegian-speaking participants, consisting of 10 men and 11 women, were recruited for the study. The participants were all right-handed, aged 21–50 years, with a mean age of 25.3 years (SD 6.2). All subjects were informed of the aim of the study and the criterions for participation. The criterions excluded people with a clear left-handed preference, people with a history of psychiatric or neurological disorders, people with claustrophobic tendencies and people with metallic implants. To control for handedness, participants filled a modified Edinburgh handedness questionnaire with 15 items, which asked for hand-preferences for certain actions, with the answering options: left, both, or right hand). All participants selected “right hand” on 12 or more items (mean 14.5 items).

The study was conducted in accordance with the Helsinki declaration, and all subjects gave written informed consent before participation per the institutional guidelines. The study was approved by the Regional Committee for Medical Research Ethics (REK-Vest).

### Paradigms

For this study, both a visual and an auditory paradigm were used. Aside from how the tasks were mediated, the conformation of the paradigms was mostly identical. Both paradigms were of a simple blocked design, with alternating experimental and control blocks. The active blocks of both paradigms contained simple tasks based on the television show “Jeopardy!” (Berntsen et al., 2006). The subjects were presented with an answer and were required to respond by generating the corresponding question. The participants were instructed to formulate their questions as a sentence, starting with the phrase “what is < target word >” A typical example used in one of the paradigms was “the color of the sky” to which the corresponding answer would be “what is blue?”. Participants were instructed not to formulate more complex sentences or sentences containing any other verbs. The participants were furthermore instructed to use covert responses in both paradigms to avoid head movements and magnetic field variations. For the control trials, participants were instructed to perceive them only attentively without any active processing. Each paradigm contained a total of 48 different tasks so that no task was repeated to the subjects at any point in the study. All stimuli were in Norwegian, and all participants were native speakers.

The visual paradigm consisted of eight active blocks, each containing six simple jeopardy-based tasks presented to the participants through MR compatible video goggles. The active blocks had a total duration of 30 s each, giving the subjects 5 s to covertly formulate an appropriate response to every task. The paradigm operated without any response data, which meant that the tasks were presented for the full 5 s before being replaced by a new task without a recorded input from the participants. The active blocks were interlaced with eight control blocks, intended to represent a baseline value without activation of the areas related to speech and language processing. These resting blocks alternated between six different rows of hashes resembling a sentence structure (e.g., #### # ## ###). These control stimuli were presented for 5 s each, as well. The combined duration of the active and the resting blocks was 8 min.

Correspondingly, the auditory paradigm was designed with eight active and eight resting blocks. The active blocks consisted of jeopardy tasks, which were similar, but not identical, to the tasks in the visual paradigm. In the auditory paradigm, the tasks were presented as pre-recorded audio files played through MR compatible headphones worn by the participants. The tasks were presented with the same 5 s frequency as in the visual paradigm. In the auditory paradigm, the resting blocks consisted of the same auditory stimuli being played backwards, rendering them unintelligible.

Both paradigms were precisely synchronized with the scanner using a synchronization box that forwarded the trigger signals from the scanner to the presentation software.

### Neuroimaging

Before entering the MRI scanner, all participants were informed about the purpose of the study and explained how the study was structured. Inside the MRI scanner, the subject’s head was padded on both sides of the headphones with additional pads to restrict movements. The visual paradigm was presented using MR compatible video goggles. The auditory paradigm was mediated through MR compatible headphones.

The structural and functional scanning was performed using a 3 Tesla General Electric Medical Systems Signa HDxt scanner. The axial slices of the functional imaging were positioned parallel to the AC-PC line with reference to a high-resolution anatomical image of the entire brain volume, which was obtained using a T1-weighted gradient echo pulse sequence. During the functional imaging, both paradigms were presented to the participants in two separate runs, but the order of the presentation was alternated for each participant. In total, 320 (2 × 190) functional images were acquired, using an T2*-weighted echo-planar imaging (EPI) sequence with the following parameter: 30 axial slices (3.4 mm × 3.4 mm × 4.4 mm) with an interleaved slice order; matrix 128 × 128; TR 3000 ms; TE 30 ms; flip angle 90°. Diffusion tensor images were collected at the end of the procedure, but these data were not used in the here presented analysis.

### Data Processing

The resulting EPI images were prepossessed and analyzed using SPM12 (v7771^1^) running under MATLAB 2016a on a Linux Ubuntu 16.04 workstation. First, the images were realigned within and between the two conditions/runs to adjust for head movements during the scanning procedure, unwarped to correct for inhomogeneity issues and controlled for remaining movement artifacts. The images were then normalized according to the stereotactic references from Montreal Neurological Institute (MNI) and resampled with a voxel size of 2 × 2 × 2 mm. Finally, the images were smoothed using an eight millimeter Gaussian kernel to reduce noise and variation between the participants.

### Head Movement

Prior to the statistical analysis, the realignment parameters of each participant were examined, with respect to total translation during the data acquisition and framewise displacement (Power et al., 2012). Only subject with less than 2 mm translation during the entire examination were considered in the further processing, and framewise displacements needed to be less than 0.5 mm.

### General Linear Model

A fixed-effect statistical model, based on the general linear model (GLM), was used as a first-level analysis on the individual datasets. Data from the two different sensory conditions were modeled according to a design matrix using the hemodynamic response as the basis function. The following contrast was defined for each participant: (1) Jeopardy task > resting block for each sensory condition separately, i.e., auditory and visual paradigm, and (2) the differences between the two conditions, i.e., visual stimuli > auditory stimuli and auditory stimuli > visual stimuli. In the second-level analysis, a group level analysis with data from all the participants was performed, resting on a one-way ANOVA model, which utilized the contrast images from the first-level analysis. The variance estimation took into account that the data were not independent, and an equal variance across paradigms was assumed. First, brain activations were explored for the two sensory modalities separately. Results were examined by applying a family-wise error (FWE) corrected threshold at the voxel level of *p* < 0.05 and at least 10 voxels per cluster.

In order to identify the areas that were significantly activated during both conditions, a “real” conjunction analysis (Nichols et al., 2005) was performed and explored with the same corrected threshold as described above.

Differences between the two tasks were explored with a difference contrast, and the same threshold was applied. Finally, the MNI coordinates were used to anatomically locate the significantly activated brain areas by using the inbuilt brain atlas of SPM12 (Neuromorphometrics^2^), by using overlays on the anatomical atlas AAL3 (automated anatomical labeling atlas, version 3; Tzourio-Mazoyer et al., 2002; Rolls et al., 2020), integrated in MRIcron^3^, and overlays on a structural MNI template, which was explored by an expert (KS).

### Intra-Class Correlation Coefficient

The main aim of the study was to explore whether results were not only comparable, as explored by the conjunction, but also reliable within individuals, across the sensory modality. Therefore, voxel-wise intra-class correlation coefficients (ICC) were estimated. An ICC is a measure of how the observed variance is split between within- and between-subject variance (Shrout and Fleiss, 1979; Fernández et al., 2003; Specht et al., 2003). Accordingly, an ICC between 0.7 and 1 is considered to indicate reasonable good reliability, since the between-subject variance is substantially higher than the within-subject variance.

### Laterality

The lateralization of the activations has been evaluated with the LI-toolbox (Wilke and Lidzba, 2007). As areas of interest served the frontal, cingulate, temporal, parietal, occipital, central, and cerebellar areas. The analysis was performed using the integrated bootstrapping method with their default values, an exclusive mask of 11 mm around the midline, and clustering. The significances of the functional asymmetries were tested for both sensory conditions independently with simple *t*-tests, and were compared across sensory conditions using paired *t*-tests. Finally, to evaluate whether the two conditions resulted in the same degree of functional asymmetry within each participant, the laterality index for the auditory condition was correlated with the laterality index of the visual condition. This was done for each of the seven areas of interest separately. Correlations across different areas were not examined.

### Between Subject Overlap

Probability maps of brain activations have been estimated. The individual *t*-maps were binarized at a threshold of *t* > 3.09 (*p* < 0.001) and summed up, using the ImCalc function in SPM12. The resulting probabilities maps were scaled to represent percent overlap of activations. These maps were explored at a threshold of at least 20% overlap (i.e., for the present study this corresponds to 4 or more subjects). This type of analysis allows identifying areas that have been consistently activated (*p* < 0.001). Second, this analysis also gives an impression to which degree areas of the right hemisphere have been activated. In other word, whether there was some variation in lateralization across subjects (Van der Haegen et al., 2011; Bishop, 2013; Hausmann et al., 2019).

### Dynamic Causal Modeling

The primary aim of this study was to reliably activate the language network through two different conditions that differed in their sensory input by keeping the task constant. Therefore, it was expected that the underlying neuronal network that is the dorsal and ventral stream would be similarly activated independently from the sensory input.

To verify this, a dynamic causal modeling (DCM) analysis (Stephan et al., 2009; Friston, 2010; Osnes et al., 2011b; Penny, 2012; Morken et al., 2017; Friston et al., 2019) was conducted using seven areas that represented the dorsal and ventral stream and that were activated in both conditions. The procedure for extracting the time series followed in the main the guidelines as described by Zeidman et al. (2019). First, the coordinates of the ROIs were determined by identifying the seven most relevant areas from the described conjunction analysis. Two sets of models were constructed, one set for the visual and one for the auditory modality. The two sets used the same seven nodes for the speech and language network, and a modality-specific eighth node as sensory input area. All DCM models were restricted to the left hemisphere and resembled the dorsal and ventral stream. The following areas were included: the superior temporal gyrus [MNI: –56 –48 10], superior temporal sulcus [MNI: –54 –12 –16], middle temporal gyrus [MNI: –52 –40 –6], frontal operculum [MNI: –46 14 –2], IFG (pars opercularis) [MNI: –48 12 26], precentral gyrus (PreCG) [MNI: –52 –6 50] and supplementary motor area [MNI: –2 10 48]. In addition, a modality-specific area served as input area for the model. This eighth node of the model was identified by the difference contrast between the two conditions, which was the visual word form area [MNI: –28 –92 0] for the visual task and the primary auditory cortex [MNI: –44 –22 2] for the auditory task. To allow for individual variability in the precise localization of the activations, time courses were extracted from the individual local maximum of activation that was less than 8mm apart from the coordinates, described above (Zeidman et al., 2019).

For each sensory modality, 57 models were defined which varied both in their underlying functional connectivity (A-matrix, 3 models) and the influence of the sensory input on the network configuration (B-matrix, 19 models). The different models are displayed in Supplementary Figure 1. A Bayesian model selection was applied for the two sensory modalities independently for identifying the specific model that expresses the detected network best (Stephan et al., 2009; Penny et al., 2010). First, the most probable family of A-models was identified and, subsequently, the respective B-model was determined. The B-models were also grouped into three families, where the sensory input mostly influenced the dorsal stream, the ventral stream, or mainly areas for perception or production, while one B-model hypothesized that the sensory input does not influence at all. The estimated parameters from the favored model for the visual and for the auditory condition were compared using paired *t*-tests.

Similar to the voxel-wise neuroimaging results, ICCs have been estimated for all DCM parameters.

## Results

### Head Movement

The largest observed head movement across an entire timeseries was 1.22 mm (group mean: 0.52 ± 0.26 mm). Further, the maximal framewise displacement across the timeseries was estimated for each participant, and the averaged maximal framewise displacement across the entire group was 0.32 ± 0.19 mm. However, three participants showed a value slightly larger than 0.5 mm. An inspection of the framewise displacements across the timeseries showed that these values originated in all three cases from a single spike for a single volume, while the values for the rest of the timeseries were below the usual threshold of 0.5 mm (Power et al., 2012). Therefore, no participant was excluded from the subsequent analyses and no motion-censoring was performed.

### Brain Activations/General Linear Model

**FIGURE 1:**
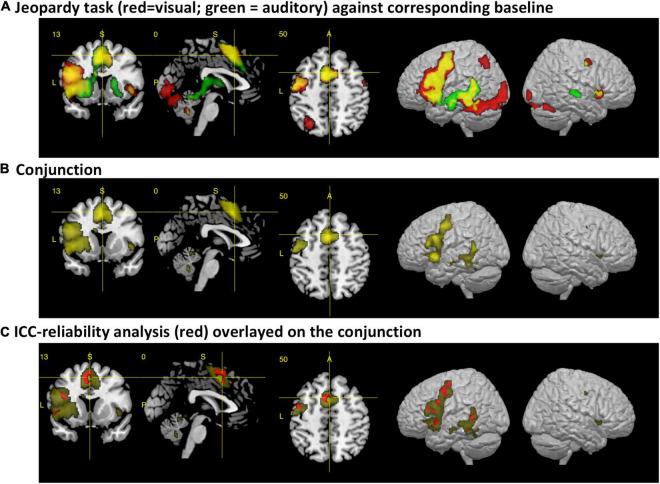
The figures display the results from the fMRI analysis with a section view and lateral render views of the left and right hemisphere. **(A)** Main activations for the visual (red) and auditory (green) variant of the paradigm after subtracting the corresponding control conditions [p(FWE) < 0.05 at voxel level, at least 10 voxels per cluster]. Activations for each condition are displayed with a voxel-wise threshold of p(FWE) < 0.05, at least 10 voxels per cluster. The areas that colored in yellow represent the additive overlap of both conditions. **(B)** Results from the *real* conjunction analysis across both paradigms [p(FWE) < 0.05, at least 10 voxels per cluster]. **(C)** Results from the cross-modal reliability analysis, using an intraclass correlation coefficient (ICC). Areas with an ICC > 0.6 are colored in red, with the conjunction analysis as background.

When compared against the visual baseline, the visual paradigm showed increased BOLD responses mostly in the areas for reading, speech perception and production of the left hemisphere, corresponding to the dorsal and ventral stream. The activated areas comprised the IFG, PreCG, aIns, supplementary motor areas (SMA), basal ganglia (BG), thalamus, hippocampus, superior temporal gyrus (STG), superior temporal sulcus (STS), middle temporal gyrus (MTG), inferior temporal gyrus (ITG), the left fusiform gyrus, lingual gyrus, and cerebellum. In the right hemisphere, the cerebellum, anterior insula, precentral gyrus, hippocampus, and brainstem were involved (see Table 1A and Figure 1A).

**TABLE 1:** The table **(A–E)** list all significant results [p(FWE) < 0.05 at voxel level, at least 10 voxels per cluster] for all estimated contrasts, and (f) the results from the analysis using an intraclass correlation coefficient (ICC > 0.6).

			MNI coordinates				Peak	Cluster
Anatomy	Side	*x*	*y*	*z*	T	p(FWE-corr)	#voxel	p(FWE-corr)
**(A) Visual**								
SMA	L	–2	10	48	15.895	0.000	1742	0.000
IFG (Oper), PreCG, IFG (Orb), BG, Thal, Hipp, aIns	L	–46	12	26	14.778	0.000	8769	0.000
ITG, FG, LinG, CalG, Cereb	L	-46	–54	–14	11.617	0.000	7773	0.000
IPL	L	–28	–62	42	11.045	0.000	744	0.000
Cereb	R	36	–58	–30	7.553	0.000	199	0.000
aIns	R	42	16	–2	7.460	0.000	372	0.000
Caudate	L	–8	10	22	7.302	0.000	21	0.004
Cereb	R	4	–56	–34	6.808	0.001	60	0.000
PreCG	R	54	–4	46	6.723	0.001	65	0.000
Hipp	R	32	–32	0	6.541	0.002	40	0.001
Amygdala	R	12	-6	–12	5.848	0.014	13	0.008
**(B) Auditory**								
SMA, IFG (Oper), PreCG, IFG (Orb), STG, MTG, BG, aIns	L	–2	10	48	15.034	0.000	11556	0.000
BG	R	18	10	10	7.956	0.000	583	0.000
STG	R	56	–18	–4	7.152	0.000	203	0.000
aIns	R	44	12	0	6.409	0.003	90	0.000
Cereb	R	4	–54	–36	6.254	0.005	33	0.002
PreCG	R	54	–4	44	6.203	0.005	18	0.005
Thal	R	20	–26	18	6.161	0.006	17	0.005
Cereb	R	36	–58	–32	5.909	0.012	11	0.010
**(C) Conjunction**								
SMA, IFG (Oper), PreCG, IFG (Orb), BG, Thal, Hipp, aIns	L	–2	10	48	15.034	0.000	1652	0.000
ITG, FG, LinG, CalG	L	–46	14	–2	10.440	0.000	5150	0.000
STG, MTG, ITG	L	–56	–48	10	8.961	0.000	1232	0.000
Brainstem	L	–6	–32	–4	8.174	0.000	527	0.000
Caudate	L	–8	10	22	7.302	0.000	18	0.005
STS	L	–54	–12	–16	6.717	0.001	46	0.001
aIns	R	44	12	0	6.409	0.003	84	0.000
Cereb	R	4	–54	–36	6.254	0.005	30	0.002
PreCG	R	54	–4	44	6.203	0.005	17	0.005
Cereb	R	36	–58	–32	5.909	0.012	11	0.010
**(D) Visual – Auditory**								
IOG, LinG, FG, Cereb	L	–28	–92	0	9.692	0.000	1838	0.000
SPL	L	–26	–60	42	6.689	0.001	47	0.001
IOG	R	24	–94	–6	6.021	0.009	39	0.001
LinG	R	16	–86	–16	5.986	0.010	116	0.000
**(E) Auditory – Visual**								
STG, HG	R	62	–20	4	8.062	0.000	218	0.000
HG, STG	L	–44	–22	2	7.894	0.000	107	0.000
Caudate	L	–60	–14	2	6.532	0.002	18	0.005
PT	L	–38	–38	10	6.270	0.004	30	0.002
**(F) ICC Analysis**					*ICC-value*			
MTG	L	–50	–46	4	*0.89*		69	
ITG	L	–48	–46	–10	*0.84*		79	
IFG (Oper), PreCG	L	–50	16	14	*0.83*		362	
SMA	L	-8	18	46	*0.81*		369	
IFG	L	–52	10	0	*0.78*		77	
aIns	L	–30	26	–2	*0.60*		13	

*SMA, Supplementary Motor Area; PreCG, Precentral Gyrus; IFG, Inferior Frontal Gyrus; IFG (Oper), Inferior Frontal Gyrus-pars opercularis; IFG (Orb), Inferior Frontal Gyrus–pars orbitalis; BG, Basal Ganglia; Caudate, Caudate Nucleus; Thal, Thalamus; Hipp, Hippocampus; aIns, anterior insula; HG, Heschl’s Gyrus; PT, Planum Temporale; STG, Superior Temporal Gyrus; STS, Superior Temporal Sulcus; MTG, Middle Temporal Gyrus; ITG, Inferior Temporal Gyrus; IPL, Inferior Parietal Lobe; FG, Fusiform Gyrus; LinG, Lingual Gyrus; CalG, Calcarin Gyrus; IOG, Inferior Occipital Gyrus; Cereb, Cerebellum.*

When compared against the auditory baseline, the auditory paradigm showed increased BOLD signals in very similar area of the left hemisphere as the visual paradigm, but with more activations within the temporal lobe and no activations within the fusiform and lingual gyrus (see Table 1B and Figure 1A).

Accordingly, the conjunction analysis showed for the central areas of the ventral and dorsal stream significantly increased brain activity for both paradigms, including the supplementary motor area, the left basal ganglia, the brainstem, the cerebellum, and the left and right anterior insula (see Table 1C and Figures 1B,C).

**FIGURE 2:**
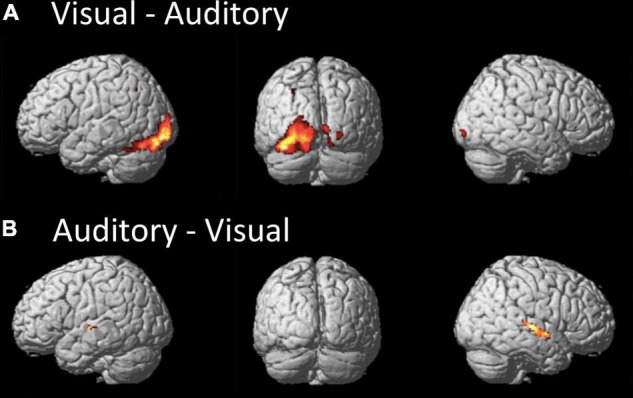
The figures display the differential effects between the visual and auditory paradigm. Results are displayed at a FWE corrected threshold of p(FWE) < 0.05 and at least 10 voxels per cluster. **(A)** Stronger activation during the visual than auditory paradigm, **(B)** stronger activation during the auditory than visual paradigm.

When exploring the differences in brain activations between the paradigms, only differential activations within areas related to the sensory processing were detected. When comparing the visual to the auditory paradigm, higher BOLD signals occurred bilaterally in the inferior occipital gyrus and lingual gyrus, and the left superior parietal lobe. Furthermore, the differential activations in the left hemisphere extended toward the area, aka the visual word form area. Interestingly, there was a strong differential effect within the left cerebellum (see Table 1D and Figure 2A).

When comparing the auditory to the visual paradigm, increased BOLD signals occurred in the left and right Heschl’s gyrus and posterior STG and STS, the left planum temporale, and the left caudate nucleus (see Table 1E and Figure 2B).

### Intra-Class Correlation Coefficient

The consistency of the activations between the sensory modalities were evaluated by estimating voxel-wise the intraclass-correlation coefficients (ICC) (Specht et al., 2003). This confirmed the observation and demonstrated that the majority of the overlapping cross-modal activations were activated to the same extent by both paradigms (ICC > 0.5, more than 10 voxels per cluster) (see Table 1F and Figure 1C). Reliable activations were found in the MTG, ITG, IFG, PreCG, and SMA.

### Laterality

All areas, except the cingulate and cerebellar area, showed a significant leftward asymmetry for both modalities, when tested separately and when a Bonferroni correction was applied (in total 21 *t*-test were performed). The cingulate area did not show any functional asymmetry and the cerebellar area demonstrated a leftward asymmetry for the visual and rightward asymmetry for the auditory paradigm. When comparing the two modalities, the results from the LI-toolbox indicated that there were significant differences only for the temporal lobe and the cerebellum, but only the difference for the cerebellum remains significant after Bonferroni correction. All other areas showed the same degree of asymmetry for both sensory modalities across the subjects (see Table 2). However, significant correlations between the condition-specific laterality indices were only found for the temporal lobe, the cingulate cortex, and central areas, but not frontal areas (see Table 2).

**TABLE 2:** The table lists the laterality index (standard deviation) for the seven examined areas.

Laterality Index	Frontal	Cingulate	Temporal	Parietal	Occipital	Central	Cerebellar
Auditory	0.641 (0.259)	0.046 (0.433)	0.650 (0.155)	0.652 (0.325)	0.307 (0.305)	0.461 (0.272)	–0.368 (0.321)
	*P* < 0.001	*P* = 0.631	p<0.001	*p* < 0.001	*p* < 0.001	*p* < 0.001	*p* < 0.001
Visual	0.666 (0.229)	0.008 (0.529)	0.737 (0.113)	0.683 (0.192)	0.369 (0.297)	0.515 (0.236)	0.211 (0.243)
	*p* < 0.001	*p* = 0.948	*p* < 0.001	*p* < 0.001	*p* < 0.001	*p* < 0.001	*p* < 0.001
Difference	*p* = 0.713	*p* = 0.569	*p* = 0.007	*p* = 0.652	*p* = 0.487	*p* = 0.371	*p* < 0.001
Correlation Auditory and Visual	*r* = 0.228 *p* = 0.321	*r* = 0.821 *p*<0.001	*r* = 0.550 *p* < 0.010	*r* = 0.397 *p* = 0.075	*r* = 0.109 *p* = 0.639	*r* = 0.434 *p* < 0.05	*r* = 0.165 *p* = 0.476

*A positive laterality index represents a leftward asymmetry. The significance of the asymmetries was tested with simple t-tests; the differences in asymmetry between the modalities were tested with paired t-tests. Only the differential asymmetry of the temporal lobe does not survive a Bonferroni correction, while all other significant effects remain significant. Further, the region-wise laterality indices were correlated between the sensory conditions.*

### Between Subject Overlap

The analysis of between-subject overlap reflects a large consistency between the subject in their activations, although only a few areas showed a 100% overlap between subjects. This was only the case for the SMA and the premotor cortex. In more than 80% of the participants, the areas of the IFG were consistently activated with a significance value of *t* = 3.09 or above. In general, the areas that were seen in the conjunction analysis also showed a good between-subject overlap. It should also be mentioned that in more than 25% of the cases, activations were detected in the right temporal lobe for the auditory condition. In almost 50% of the cases, right frontal activations were detected during the visual condition.

### Dynamic Causal Modeling

**FIGURE 3:**
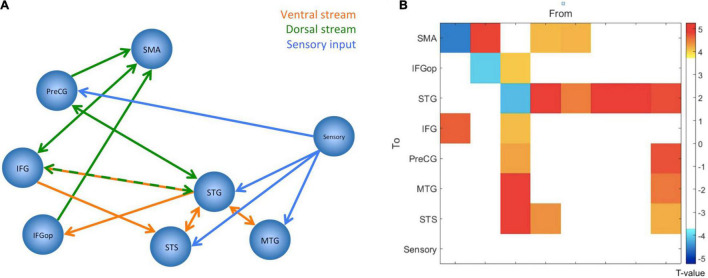
The figure summarizes the results from the dynamic causal modeling analysis. Since there were no significant differences between paradigms, parameters have been averaged, and the figure displays the network configuration after one-sample *t*-tests and Bonferroni correction. **(A)** The figure displays the network configuration. All displayed connections are excitatory, but self-inhibitory connections are not displayed. The blue lines indicate the connections from the sensory area into the speech and language network, the orange lines should illustrate the ventral stream, and the green lines should illustrate the dorsal stream. **(B)** The same result as connectivity matrix. The colors represent the *T*-values for the connections, as indicated by the color bar to the right. The node “Sensory” corresponds to the visual word form area for the visual paradigm and the primary auditory cortex for the auditory paradigm. SMA, Supplementary Motor Area; PreCG, Precentral Gyrus; IFG, Inferior Frontal Gyrus; IFGop, Inferior Frontal Gyrus-pars opercularis; STG, Superior Temporal Gyrus; STS, Superior Temporal Sulcus; MTG, Middle Temporal Gyrus.

The DCM results were explored – separately for the two paradigms – in a hierarchical process. First, a Bayesian model selection (BMS) was applied to the three A-model families that varied in their underlying connectivity matrix (see Supplementary Figure 1a). This revealed the fully connected model for both modalities. Then the BMS was applied to the remaining 19 models that varied according to their B matrix (see Supplementary Figure 1b). This revealed no modulations of connection (the “null model”) in the visual paradigm, but increased connectivity between the IFG and the frontal operculum in the auditory paradigm. When exploring the estimated parameter with a one-sample *t*-test, however, this parameter appeared not to be significant (*p* = 0.07). Therefore, only the A-matrix was statistically compared between the two modalities. This was done by performing 64 paired *t*-tests (i.e., an 8 × 8 A-matrix for both modalities) but after applying a Bonferroni correction, there were no significant differences between the two A-matrices. Therefore, it was concluded that there were no systematic differences in the A-matrices between the two modalities. Accordingly, the two A-matrices were averaged for the analysis of the involved neuronal network. The averaged A-matrix was re-examined with 64 one-sample *t*-tests, and, accordingly, a Bonferroni correction was applied. This revealed a network of significant connections that mostly resampled the dorsal and ventral stream (see Figure 3).

Although there were no significant differences between the A-matrices of the two paradigms, all 64 elements were subjected to an ICC analysis. The results revealed that the ICC values were lower than for the voxel-wise analysis described above (all ICCs < 0.4).

## Discussion

The overall aim of this study was to develop a short language paradigm for clinical applications that would reliably activate central parts of the speech and language network, and that would give identical results independent whether stimuli are presented visually or aurally. For the present study a “Jeopardy!” paradigm was selected since it was expected that this paradigm would involve several core language processes, such as lexical, semantic, and syntactic processing (Berntsen et al., 2006). These functions are mostly covered by both the dual-stream (Hickok and Poeppel, 2004, 2007) and the extended dual-stream model (Specht, 2014), and it was hypothesized that this paradigm would confirm these models both in terms of activations and connectivity.

### Brain Activations

As expected, both paradigms activated (when the corresponding sensory control condition was subtracted) a widespread network of left temporal and frontal areas (see Figures 1A,B). The visual paradigm largely resembles the activations found by the original study (see Figure 1; Berntsen et al., 2006). In accordance with the extended dual-stream model (Specht, 2014), activations were also detected within the basal ganglia and anterior insular cortex. While there was an activation of the right anterior insular cortex for both paradigms, activation of the right superior temporal sulcus was only observed for the auditory paradigm (see also Figure 2).

This is also reflected by the results from the analysis of the functional asymmetry that indicated differences between the two paradigms for the temporal lobe, but not for the frontal lobe. Although both paradigms showed a significant leftward asymmetry, the additional activation of the right superior temporal sulcus gave a significantly weaker leftward asymmetry for the auditory paradigm (see Table 2). However, the effect becomes non-significant when a Bonferroni correction is applied.

Interestingly, the visual paradigm apparently activated the left cerebellum stronger than the auditory paradigm. This was also confirmed by the analysis of functional asymmetry that demonstrated differential asymmetry only for the cerebellum but not occipital lobe (see Table 2 and Figure 2A). The stronger involvement of the cerebellum supports the notion that the cerebellum might be more involved in reading due to a required translation into phonological codes, which is not necessary for the processing of auditory information, while the cerebellar functional asymmetry of language processes is still controversially discussed (Mariën et al., 2013).

In general, there was a considerable consistency between the two paradigms, not only when comparing activation patterns, but also when analyzing them with a conjunction analysis and an analysis of the reliability of activations across paradigms. Both paradigms involved most of the hypothesized areas, and the ICC analysis demonstrated that the posterior STG and STS, MTG, frontal operculum, IFG, precentral gyrus, and SMA showed high reliability of the activation strength. These are important core areas of the speech and language network (Price, 2012; Specht, 2014). However, against our *a priori* hypothesis, the inferior parietal lobe, in particular the angular gyrus, and the temporal pole were not activated by this task – even not at a reduced significance threshold. This may indicate that this task activates the central parts with the most basal functions of the speech and language network, but the semantic and syntactic system is not activated to the full extend. One reason for that might be that the requested response sentences are very simple and were of the type “what is < target word >” and did not contain any complex syntax or other verbs.

**FIGURE 4:**
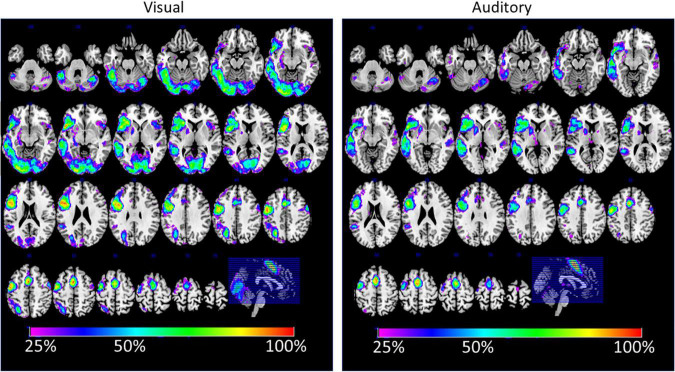
The figure displays the percent-wise overlap of brain activations for the visual **(left)** and auditory **(right)** sensory condition. The estimation is based on the individual spmT-maps, which were binarized at a threshold of *T* > 3.09 (corresponding to *p* < 0.001).

Further, a detailed analysis of the functional asymmetry revealed that, although the overall group results were comparable, there were different degrees of functional asymmetry between the sensory conditions. More importantly, the laterality indices for the functional asymmetry correlated only for the cingulate, central, and temporal regions but not for the frontal lobe. This indicates that, for the frontal lobe, the individual degree of functional asymmetry was different between the auditory and visual condition (see Table 2). This can also be seen in the analysis of overlapping activations (see Figure 4, left). Here, the right frontal lobe seems to be activated during the visual condition in some participants, as there is overlapping activations in about 25–30% of the participants, while the same area was inactive for the auditory condition (see Figure 4, right). Although this activation did not become significant on the group level, it might indicate that there exists some individual variability within the right frontal lobe, and, more interestingly, that this area might become active only during reading but not listening. Thereby, an individually estimated functional asymmetry index might give different results for a reading and a listening task, while there was more consistency for the temporal lobe.

The results demonstrate that the two sensory conditions equally activated the areas of the left temporal lobe. Central parts for the comprehension of a language message are the posterior parts of the STG, STS, and MTG. These are the areas that are mostly associated with word processing and semantic decoding (Price, 2012). The left STS plays a particular function in this network, as it is not only a multisensory area (Specht and Wigglesworth, 2018), but also an area that is very sensitive to phonetic information in an acoustic signal, as demonstrated by the “sound morphing” paradigms (Specht et al., 2005, 2009; Osnes et al., 2011a). Later, Sammler et al. (2015) could demonstrate with a similar paradigm a corresponding effect for prosody processing for the right STS. This study also reflects this differential responsiveness and division of labor between the left and right STS since both paradigms activate the left STS, but only the auditory variant activates the right STS. This is further confirmed by the difference contrast between the paradigms that mostly showed differences for the right STS (see Figure 2B).

The other areas that were detected by this study can mainly be associated to lexical processing and sentence generation (Heim et al., 2009; Grande et al., 2012) and the generation of the (covert) response (Eickhoff et al., 2009; Price, 2012). Referring to the conjunction analysis, these areas comprise the IFG, frontal operculum, PreCG, SMA, but also the subcortical areas aIns and BG. While the contribution of the cortical areas in speech processes have been demonstrated by many studies before, these findings supplements the ongoing discussion that models of speech and language functions also have to include subcortical areas (Specht, 2014). There is an increasing awareness of various speech, language, and speech motor function of the aIns (Dronkers, 1996; Eickhoff et al., 2009; Ackermann and Riecker, 2010; Nieuwenhuys, 2012; Rodriguez et al., 2018). The same is true for the basal ganglia with discussed involvements in both perception and production (Eickhoff et al., 2009; Kotz et al., 2009; Enard, 2011; Lim et al., 2014; Kotz and Schmidt-Kassow, 2015). However, since the current paradigm involves both the perception and production, it is not a suitable paradigm for disentangling the specific function of these subcortical areas or anatomical subdivision to certain processes.

However, the paradigm was not efficient enough to activate the semantic and syntactic processing network to its full extend, since the angular gyrus, supramarginal gyrus, and temporal pole were not detected – even not at an uncorrected threshold. As aforementioned, the reason for this might be that this task is semantically and syntactically too simple since only everyday knowledge is requested by the task and neither the stimuli nor the responses are syntactically complex and always of the same type. The lack of activation in the anterior temporal lobe could be explained through the observations in patients with primary progressive aphasia of the semantic type. These patients show focal atrophy of the anterior temporal lobe causing predominantly semantic deficits (Zahn et al., 2005; Wilson et al., 2014). More specific, Wilson and colleagues could demonstrate that this degeneration seems to affect more the higher-level processing rather than simple syntactic processes (Wilson et al., 2014). Further, the activations within the semantic network might vary dependent on the processed category, amount of lexical information, or the requested semantic relation (Patterson et al., 2007; Binder et al., 2009; Graessner et al., 2021). Although the angular gyrus and its subdivisions are often involved in semantic processing at various levels of complexity, the angular gyrus needs to be seen as a part of a broader network with strong fronto-parietal interactions (Graessner et al., 2021), where at least some of the other nodes, like the anterior IFG, got activated by both tasks.

Eventually, a clinical paradigm that aims to activate not only the core areas but the speech and language system to its almost full extend should seek for a balance between a task that is easy enough to be perform by impaired patients but with a higher degree of syntactical, lexical, semantic and combinatory complexity and well-controlled semantic categories and semantic relations. Further, one might consider a way of actively controlling the performance of the participants to overcome the lack of overt responses through random catch trials which require an additional response through button presses. Those performance control might also stabilize the activation patterns in the detected network and might also improve the signal in those areas that were not reliably detected.

### Dynamic Causal Modeling

The discovered core areas served as input areas for the DCM analysis, which was initially independently analyzed for the two paradigms. Importantly, the subsequent analyses revealed that there were no significant differences in connectivity between the two paradigms. However, the reliability of the estimated parameter was much lower than for the voxel-wise ICC analysis, which corresponds to earlier observations (Frässle et al., 2015). This may indicate that voxel-wise analyses are currently more stable than DCM based analyses – at least for one of the two sensory modalities. This may be an important aspect and limitation for future clinical application of network-based analysis methods.

Since there were no systematic differences between the paradigms, the estimated DCM parameters were averaged and jointly analyzed for the two paradigms. The absence of significant differences may further indicate that the sensory modality of the original stimuli does not influence the information flow within the given network.

Importantly, the dual-stream model nicely emerged from this analysis. According to the DCM results, the posterior STG and the IFG serve as central nodes of this network, with several forwards and backwards connections. The sensory information enters this network at all nodes of the temporal lobe and, interestingly, the PreCG. This latter aspect might give some further evidence to the ongoing discussion of direct motor involvement during speech perception (Liberman and Mattingly, 1985; Galantucci et al., 2006; Devlin and Aydelott, 2009; Osnes et al., 2011b). The current study indicates that also reading may involve motor areas. However, an in-depth discussion of this controversy is out of the scope of the present report.

The most obvious result from the DCM analysis is that the network structure nicely represents the ventral and dorsal streams (see Figure 3A). The high degree of bidirectional connectivity between the nodes of the temporal lobe may resemble the ventral stream. This is contrasted by a mostly frontal connectivity pattern that cumulates in more forward connections to the SMA. This pattern might resemble the dorsal stream and is, therefore, crucial for the production of the speech sounds. The SMA is an important area for generating motor responses (Nachev et al., 2008), but has also been linked to auditory processing and auditory imagery (Lima et al., 2016). Since the participants performed covert responses, the seen activations might be a mixture of motor preparation and auditory imagery. As can be seen in Figure 1, the activation involves both pre-SMA and SMA (Nachev et al., 2008) with the most significant spot within SMA. Notably, the ICC analysis indicated reliable activations in both parts of the SMA (see Figure 1C).

The posterior STG appears to be the node with the highest degree of connectivity and as the connecting node between the two hypothesized streams since there are bidirectional connections of the STG, not only to the other nodes within the temporal lobe, but also to the IFG, and PreCG, and a forward connection to the frontal operculum (see Figure 3). This is in accordance with current models that postulate a similar connectivity pattern of the posterior temporal areas as an area that connects the two streams (Hickok and Poeppel, 2007; Rauschecker and Scott, 2009; McGettigan and Scott, 2012; Specht, 2013, 2014; Binder, 2016). However, the used paradigm is not suitable to disentangle the different functions of the various connections in the comprehension and production process. Given the detected pattern, it is nonetheless evident that the frontal areas are mostly dedicated to the production since the ultimate endpoint of the connections is the SMA, which is the central area for planning and execution of motor actions.

### Reliability and Future Clinical Application

Besides the general activation and connectivity patterns that were elicited by the two paradigms, it was also examined whether these patterns were replicable between the two sensory modalities. This was achieved by supplementing the analysis with an ICC analysis that tested whether the detected strength of the activations and the parameter of the effective connectivity were replicable, independent of the sensory input modality. Since the paradigm aimed to identify the speech and language network equally well by both modalities, this is an important measure prior to future clinical applications.

Comparing the conjunction and ICC analysis, the conjunction was more extended. This is mostly caused by the fact that the conjunction analysis highlights all areas that were significantly activated by both paradigms (Nichols et al., 2005), while the ICC analysis answers a much stronger question, namely which areas showed the *same* level of activation in both paradigms (Specht et al., 2003). It is important to emphasize that, although the areas that showed reliable levels of activations are much smaller than those indicated by the conjunction, all important nodes of the dual-stream model are included (Poeppel and Hickok, 2004; Hickok and Poeppel, 2007; Specht, 2013, 2014). This is an inevitable prerequisite for a clinical paradigm that aims to examine the speech and language network with only one type of paradigm that is independent of the sensory stimuli of the to be processed stimuli.

The ICC analysis of the estimated parameter from the DCM analyses indicated that the estimation of the parameters were more variable between the two paradigms. Although they were not significantly different between the two paradigms, the distribution of the within- and between-subject variance was inferior to the voxel-wise analysis. Consequently, a DCM analysis may be a useful tool for examining underlying network structures *per se*, but voxel-wise analyses appear to be superior in a clinical context – at least for the given paradigm. However, an expanded DCM analysis, which covers both hemisphere might be an even more efficient approach, despite computational limitations.

Irrespective of that, before such a paradigm could be routinely used in a clinical setup, it needs further replication in different population and across scanner (and software) platforms. This is especially of relevance in the light of the “replication crisis” (Maxwell et al., 2015).

Similarly, an examination of the effects of motion on the results and the explored network structure goes beyond the scope of the current report. However, this is an important aspect in the context of clinical fMRI where head movements are a substantial source of noise, and which needs to be examined further.

## Conclusion

The results demonstrate that, independent from the sensory modality, this paradigm reliably activated the same brain networks, namely the core areas of the dorsal and ventral stream for speech processing. Only the cerebellum demonstrated differential effects. Further, the ICC analysis revealed that there was high reliability of brain activation across sensory modalities. This was supported by the fact that the DCM analysis showed that the underlying network structure and connectivity was the same between sensory modalities, although the parameter of the effective connectivity appear to vary with the sensory modality. However, a closer inspection of the individual functional asymmetry indicated that the degree of functional asymmetry was not the same for the two conditions. In particular, the visual condition showed in more individuals a right frontal activation than the auditory condition. This effect needs further exploration.

In conclusion, the explored paradigm activated the most central parts of the speech and language network, mostly independently of whether the stimuli were administered aurally or visually. Further, the DCM analysis revealed that the underlying connectivity patterns of the left hemisphere were similar, if not identical, for both paradigms, although the reliability was lower than for the activation data. Taking both aspects together, this paradigm appears to be suitable as a clinical paradigm since both patients with visual or aural disabilities can be equally examined. However, to stimulate the speech and language system more complete, one might better use syntactically and semantically more complex stimuli since the current paradigm could reliably activate only the most central core areas. Further, before this paradigm is ready for a broader clinical application, one needs to replicate these findings in an independent sample, which were examined on a different MR scanner. Further, the degree of leftward asymmetry, and its inter-individual variability, needs further critical examination. Third, the applicability of the paradigm in congenital or acquired deaf or blind people should be evaluated. Nevertheless, the provided evidence let one assume that this type of paradigm is highly suitable for various clinical applications.

## Data Availability Statement

The datasets presented in this article are not readily available because data sharing has been restricted through the evaluation of the ethical committee. Requests to access the datasets should be directed to KS, Karsten.specht@uib.no.

## Ethics Statement

The studies involving human participants were reviewed and approved by Regional Ethical Committee – West, University of Bergen. The patients/participants provided their written informed consent to participate in this study.

## Author Contributions

ER, KM, and KS designed the study and analyzed the data, based on the GLM. ER and KM created and recorded the stimuli, collected the data. KS performed the DCM and asymmetry analyses. ER and KS wrote the manuscript. All authors contributed to the article and approved the submitted version.

## Conflict of Interest

The authors declare that the research was conducted in the absence of any commercial or financial relationships that could be construed as a potential conflict of interest.

## Publisher’s Note

All claims expressed in this article are solely those of the authors and do not necessarily represent those of their affiliated organizations, or those of the publisher, the editors and the reviewers. Any product that may be evaluated in this article, or claim that may be made by its manufacturer, is not guaranteed or endorsed by the publisher.

## Footnotes

1
http://fil.ion.ucl.ac.uk/spm

2
http://www.neuromorphometrics.com

3
http://nitrc.org/projects/mricron
