# Supplementary material for: Evaluation of a Simple Clinical Language Paradigm With Respect to Sensory Independency, Functional Asymmetry, and Effective Connectivity
Source: Front Behav Neurosci. 2022 Mar 3;16:806520. doi: 10.3389/fnbeh.2022.806520 (PMC8928437; doi:10.3389/fnbeh.2022.806520)
Supplement: Supplementary file 1 [file Data_Sheet_1.PDF]

Supplementary Figures

Figure S1: Display of the different DCM models

a) A-models

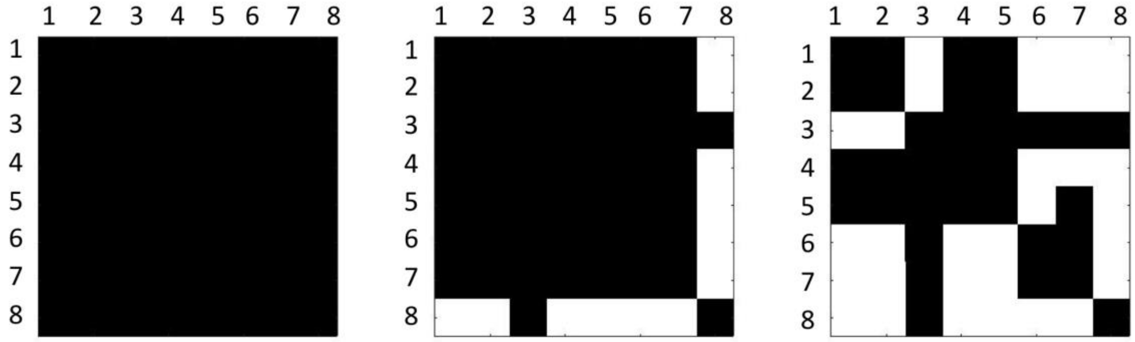

The order of the areas is:

1=SMA/Supplementary Motor Area

2=IFGop/Inferior Frontal Gyrus-pars opercularis

3=STG/Superior Temporal Gyrus

4=IFG/Inferior Frontal Gyrus

5=PreCG/Precentral Gyrus

6=MTG/Middle Temporal Gyrus

7=STS/Superior Temporal Sulcus

8=Sensory (depending on the paradigm this was either the visual word form area (VWFA) or left primary auditory cortex (pAC))

b) B-Models (except null-model)

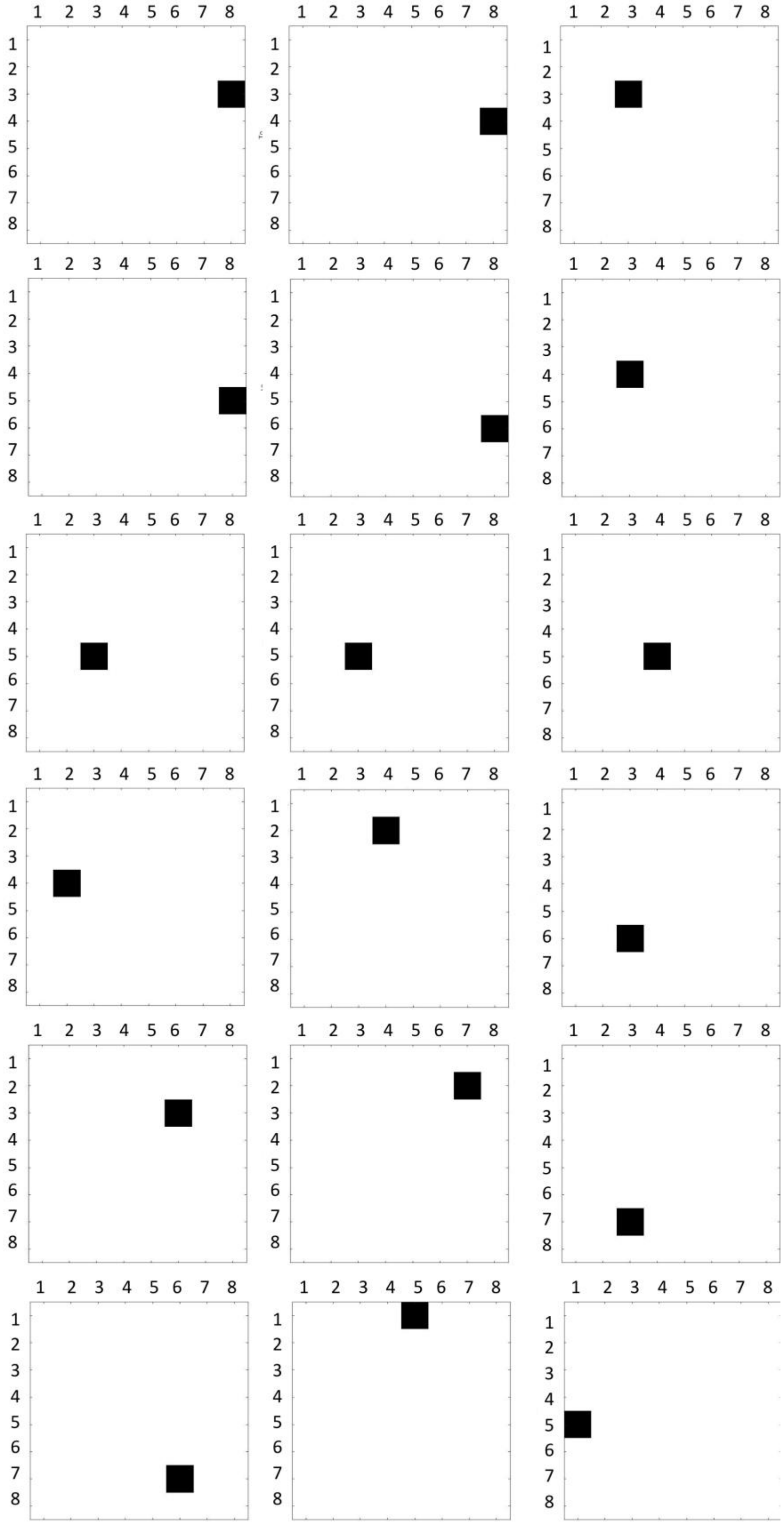

The order of the areas is:
1=SMA, 2=IFGop, 3=STG, 4=IFG, 5=PreCG, 6=MTG, 7=STS, 8=Sensory (VWFA or pAC)
